# Exogenous ethephon treatment on the biosynthesis and accumulation of astragaloside IV in *Astragalus membranaceus* Bge. Var. *Mongholicus* (Bge.) Hsiao

**DOI:** 10.1186/s40529-024-00426-y

**Published:** 2024-07-05

**Authors:** Haonan Wu, Hang Cui, Yu Tian, Jiawen Wu, Zhenqing Bai, Xiujuan Zhang

**Affiliations:** 1 College of Life Sciences, Yan’an University, Yan’an, 716000 China; 2 Inner Mongolia Academy of Science and Technology, Hohhot, 010018 China

**Keywords:** *Astragalus Membranaceus* Bge. Var. *Mongholicus* (Bge.) Hsiao, Ethephon, Astragaloside IV, Gene expression

## Abstract

**Background:**

Astragaloside IV is a main medicinal active ingredient in *Astragalus membranaceus* Bge. var. *mongholicus* (Bge.) Hsiao, which is also the key biomarker of *A. membranaceus* quality. Ethylene has been well-documented to involve in secondary metabolites biosynthesis in plants. Nevertheless, how ethylene regulates astragaloside IV biosynthesis in *A. membranaceus* is still unclear. Therefore, in the present study different dosages and time-dependent exogenous application of ethephon (Eth) were employed to analyze astragaloside IV accumulation and its biosynthesis genes expression level in hydroponically *A. membranaceus*.

**Results:**

Exogenous 200 µmol·L^− 1^Eth supply is most significantly increased astragaloside IV contents in *A. membranaceus* when compared with non-Eth supply. After 12 h 200 µmol·L^− 1^ Eth treatment, the astragaloside IV contents reaching the highest content at 3 d Eth treatment(*P* ≤ 0.05). Moreover, After Eth treatment, all detected key genes involved in astragaloside IV synthesis were significant decrease at 3rd day(*P* ≤ 0.05). However, *SE* displayed a significant increase at the 3rd day under Eth treatment(*P* ≤ 0.05). Under Eth treatment, the expression level of *FPS*, *HMGR*, *IDI*, *SS*, and *CYP93E3* exhibited significant negative correlations with astragaloside IV content, while expression level of *SE* displayed a significant positive correlation.

**Conclusions:**

These findings suggest that exogenous Eth treatment can influence the synthesis of astragaloside IV by regulating the expression of *FPS*, *HMGR*, *IDI*, *SS*, *CYP93E3* and *SE*. This study provides a theoretical basis for utilizing molecular strategies to enhance the quality of *A. membranaceus*.

**Supplementary Information:**

The online version contains supplementary material available at 10.1186/s40529-024-00426-y.

## Background

*Astragalus membranaceus* Bge. var. *mongholicus* (Bge.) Hsiao (*A. membranaceus*) is a traditional Chinese medicinal (TCM) herb renowned for its tonifying properties, diuretic effects, cerebral ischemic diseases and immune system disorders medicinal efficacy (National Pharmacopoeia Committee 2020; Hu et al. 2021; Kong et al. 2018; Tsai et al. 2019). However, the quantity of *A. membranaceus* yield in the field always fails to meet the demands required by the market (Liu et al. 2020). Furthermore, the quality of *A. membranaceus* in the field faces the problem that active ingredients contents are easily decreased by unfavorable environmental factors (Yang et al. 2022; Chen et al. 2022). To address this issue, hydroponics, a soilless cultivation method, has been increasingly concerned as its shorter growth cycles, high yields, improved quality, and reduced susceptibility to pests and diseases merits (Barrett et al. 2016). Hydroponics has been extensively employed in the organic production of various Chinese medicinal herbs, including dandelion, ligularia, peppermint, and ginseng (Minling et al. 2022; Wei et al. 2018; Ćavar Zeljković et al. 2022; Lee et al. 2022). Moreover, it has emerged as a key approach for the cultivation of Chinese medicinal herbs in recent years. Our group have indicated that Astragaloside IV contents in *A. membranaceus* grown hydroponically are remarkably higher than that in filed growth (Chen et al. 2021).

Astragaloside IV is a major active ingredient in *A. membranaceus* and deploys a tetracyclic triterpenoid saponin structure (Li et al. 2017). The biosynthetic pathway of tetracyclic triterpenoid saponins primarily involves the mevalonic acid pathway located in the cytoplasm (Chen et al. 2015). These genes identified in the pathway can be categorized into three groups: (1) upstream genes involve in isopentenyl diphosphate (IPP) and dimethylallyl diphosphate (DMAPP) biosynthesis, including acetoacetyl-cozymeA (CoA) thiolase (AACT) and 3-hydroxy-3-methyl glutaryl coenzyme A reductase (HMGR). These genes directly catalyze the generation of mevalonate (Tian et al. 2015); (2) the genes involve in the formation of terpene carbon skeletons and intermediates, including squalenesynthase (SS), squaleneepoxidase (SE) and cycloartenolsynthase (CAS). These genes catalyze the production of 2, 3-oxidosqualene leading to triterpene skeleton cyclization (Yoshioka et al. 2020); (3) downstream genes involve in complex structural modifications of intermediates and terpene compounds, including cytochrome P450 (CYP450) and UDP-glycosyltransferases (UGT). These genes play a role in modifying the triterpene skeleton, leading to the synthesis of various triterpene compounds (Dai et al. 2015; Wang et al. 2020).

Ethylene is a regulatory molecule in plants as well as regulating plant secondary metabolites synthesis (Zhao et al. 2005; Shahrajabian et al. 2022). For instances, ethylene boosts secondary metabolite production especially total flavonoid levels in sandalwood leaves (Li et al. 2021). Ethylene promotes saponin accumulation by upregulating key genes encoding squalene synthase and squalene epoxidase in squalene biosynthesis. Application of ethylene precursor, 1-aminocyclopropane-1-carboxylic acid (ACC) enhances saponin contents in ginseng cells (Rahimi et al. 2015). Ethylene treatment elevates the total saponin contents in young Gynostemma leaves (Xu et al. 2020). Wang et al. (2022) found that spraying ethephon on “Fujiminori” grape ears before color change can up-regulate anthocyanin synthesis structural genes such as *VvPAL*, *VvC4H VvUFGT*, and anthocyanin synthesis regulatory gene *VvMYBA1*, the expression of *VvMYBA2* accelerates the accumulation of anthocyanins in grapes. Ni et al. (2021) found that ethylene induced *PpERF105* inhibits anthocyanin biosynthesis by upregulating *PpMYB140* expression. Nevertheless, regulation of ethylene on triterpenoid saponin biosynthesis in *A. membranaceus* is still unclear.

To reveal how ethylene regulates astragaloside IV biosynthesis and accumulation in *A. membranaceus* roots, *A. membranaceus* plants were treated with different dosages and time of exogenous ethylene grown hydroponically. The contents of astragaloside IV and the expression levels of relevant key genes including *AACT*, *HMGS*, *HMGR*, *IDI*, *FPS*, *SS*, *SE*, *CAS*, *CYP88D6* and *CYP93E3* were determined.

## Materials and methods

### Plant materials and growth conditions

Hydroponic experiments were conducted at the Inner Mongolia Institute of Science and Technology, Hohhot City, Inner Mongolia Autonomous Region (31°24′N, 121°29′E), Northwest China. Seeds were surface-sterilized and then sown in a ratio mixture of 1: 3 of vermiculite with commercial nutrient soils (Xiangzheng Agriculture Technology, Hunan). After germination, the seedlings were thinned for healthy and strong growth. The *A. membranaceus* plants were cultured a controlled growth room with a photoperiod of 16 h at 25 °C and 8 h darkness at 22 °C. After 30 d cultivation, uniform seedlings of *A. membranaceus* were selected for hydroponics.

The 30-day-old uniform *A. membranaceus* seedlings were transferred into nutrient solution consisting of 5 mM KNO_3_, 2 mM Ca (NO_3_)_2_, 2 mM MgSO_4_, 2.5 mM KH_2_PO_4_, 14 µM MnCl_2_, 70 µM H_3_BO_3_, 1 µM ZnSO_4_, 0.5 µM CuSO_4_, 0.2 µM Na_2_MoO_4_, 0.01 µM CoCl_2_, 50 µM Fe-EDTA, and 4.7 mM MES buffer. After 7 d pre-cultivation, different concentrations of ethephon 0 µmol·L^− 1^, 50 µmol·L^− 1^, 200 µmol·L^− 1^ and 500 µmol·L^− 1^ were added into nutrient solution, collect samples at 0 h, 12 h, 3 d, 7 d. Roots of *A. membranaceus* after different dosages of ethephon application were washed and dried at 45 °C until constant weight. Contents of astragaloside IV in roots treated with different dosages of ethephon were determined.

Based on the contents of astragaloside IV, the dosage of 200 µmol·L^− 1^ ethephon was selected for further studying the time-dependent effects of ethephon on astragaloside IV contents. The growth conditions of *A. membranaceus* seedlings were the same as that in different dosages of ethephon application experiment. After 7 d pre-cultivation, 200 µmol·L^− 1^ ethephon were added into nutrient solution, while non- ethephon addition was taken as the control. The samples were harvested at 0 h, 12 h, 3 d and 7 d after treatment. One part of the roots was dried at 45 °C until constant weight for astragaloside IV determination. Another part of roots was frozen in liquid nitrogen and then stored at -80 °C for genes expression determination.

### Determination of astragaloside IV

The detection of Astragaloside IV by HPLC was conducted on a 1260 Infinity II Prime LC System (Agilent Technologies, Beijing, China) which is composed of a 1260 Infinity II Diode Array Detector HS, 1260 Infinity II Multisampler, and a 1260 Infinity II Binary Pump. A reverse phase C18 column, Agilent Zorbax sb-c18 column (4.6 × 250 mm), was used at 27 °C. The mobile phase was acetonitrile water (32: 68), the flow rate was 1.0 ml·min^− 1^. the ELSD parameters are as follows: evaporator temperature: 112 °C, nebulizer temperature: 85 °C, gas flow rate: 1.5 SLM, data rate: 80 Hz, led intensity: 100%, smoothing: 50 (5.0 s), and PMT gain: 10.0; the theoretical number of astragaloside IV was not less than 4 µl, 10 µl, 20 µl, and 10 µl of the reference solution, and the test solution was, respectively, injected into the liquid chromatograph. The determination was carried out according to the above chromatographic conditions, and the HPLC liquid chromatograms were recorded (Chen et al. 2021).

### RNA extraction and quantitative real-time PCR determination

Total RNA of *A. membranaceus* roots were extracted according to the instructions of the Total RNA extraction kit (TAKARA, Japan). The RNA was reversedinto cDNA by using the PrimeScript™ RT reagent Kit with gDNA Eraser (Perfect Real Time) kit (TAKARA, Japan). The relative expression levels of *AACT*, *HMGS*, *HMGR*, *IDI*, *FPS*, *SS*, *SE*, *CAS*, *CYP88D6* and *CYP93E3* were determined based on 95 °C for 30 s, 95 °C for 5 s, 60 °C for 30 s, 40 cycles by using a real-time PCR machine (Applied Biosystems, Thermo Scientific, America). 18 S was used as the internal reference gene. The three biological replicates were determined for each sample. The gene expression levels were analyzed using the 2^−ΔΔCt^ method.

### Data analysis

The statistical analysis for all the data were conducted using software Excel 2010 and SPSS software (version 20.0). All the data were presented as the mean values ± standard deviations of three replicates. The significance differences were determined according to student t test at the level of 0.05 and 0.01. Graphs were generated using Origin 2021.

## Results

### The accumulation of astragaloside IV is affected by exogenous ethephon treatment in *A. membranaceus*

**Fig. 1:**
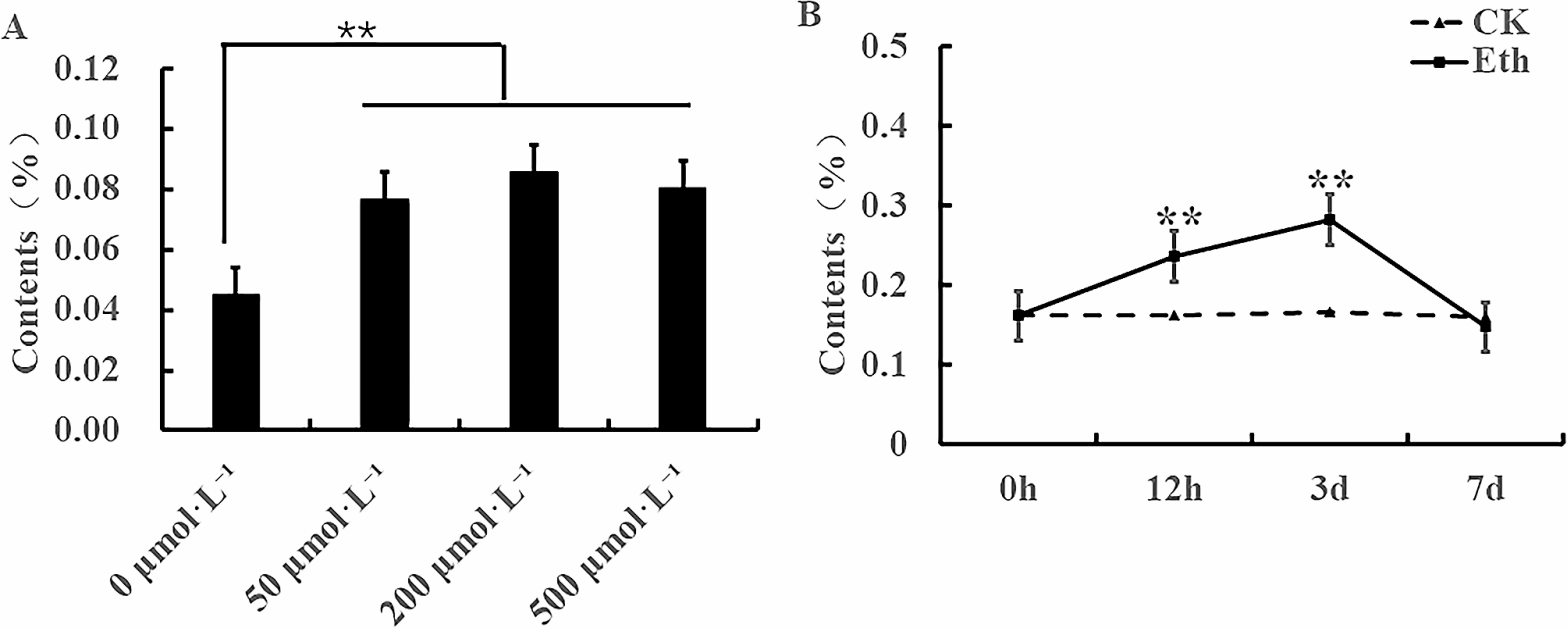
Effects of different dosages of ethephon on the contents of astragaloside IV **(A)**. Effects of different time treatments with 200 µmol·L^− 1^on the contents of astragaloside IV **(B)**. “**” represents the significant differences between control and the corresponding ethephon treatment at the level of 0.01

To investigate whether astragaloside IV contents in *A. membranaceus* were in an ethylene-dependent manner, exogenous 0 µmol·L^− 1^, 50 µmol·L^− 1^, 200 µmol·L^− 1^ and 500 µmol·L^− 1^ ethephon (Eth) were applied. Compared with non-Eth addition, exogenous application of Eth all significantly increased astragaloside IV contents in roots of *A. membranaceus* (Fig. 1A). Furthermore, *A. membranaceus* were treated with 200 µmol·L^− 1^ Eth after 0 h, 12 h, 3 d and 7 d. Astragaloside IV contents in non-Eth-treated roots of *A. membranaceus* were not changed as the time increased from 0 h to 7 d. By contrast, astragaloside IV contents in roots of *A. membranaceus* were remarkably enhanced by 200 µmol·L^− 1^ Eth after 12 h and 3 d treatments, while the astragaloside IV contents reached the peak after 3 d Eth treatments (Fig. 1B).

**Fig. 2:**
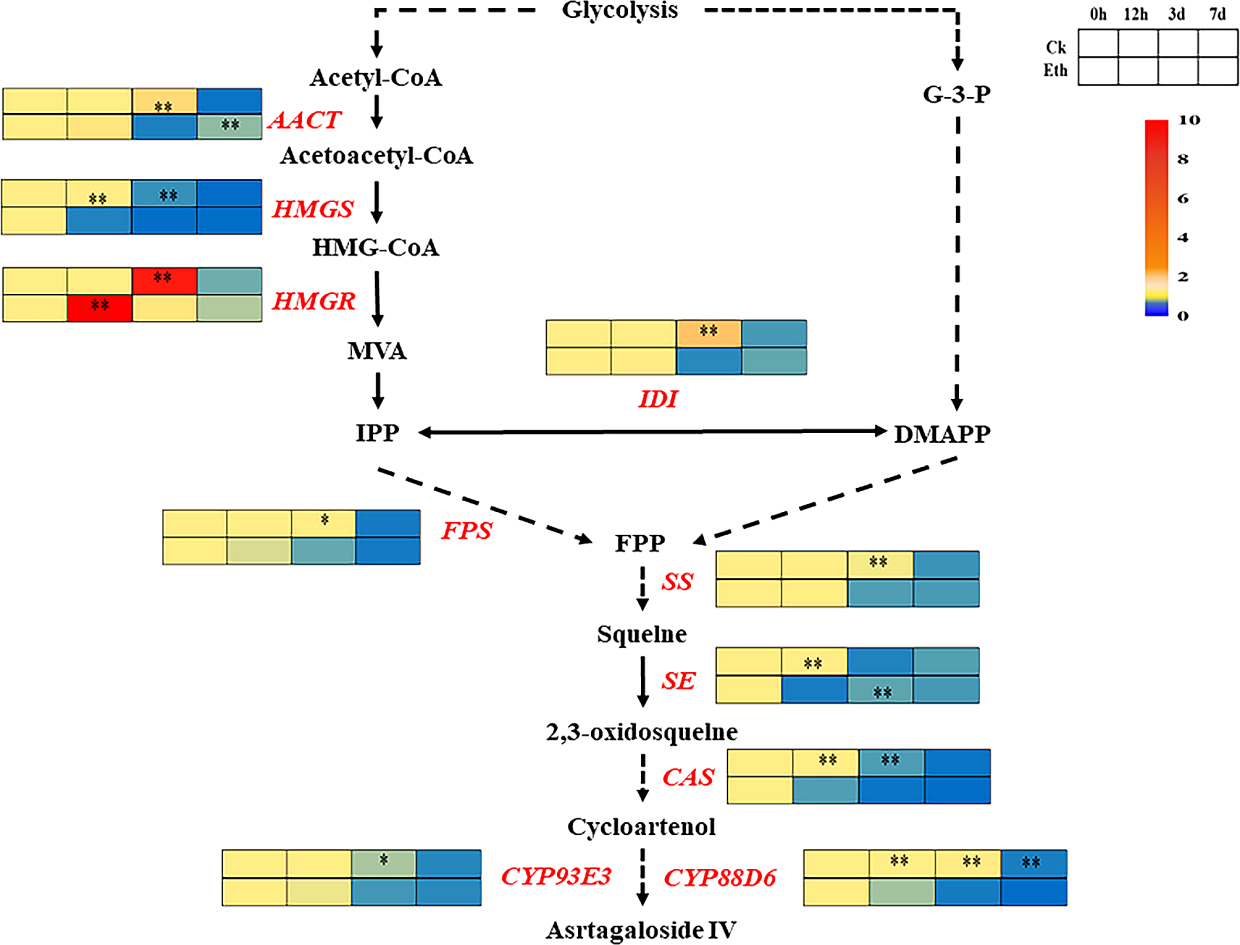
Effects of 200 µmol·L^− 1^ ethephon on the ten key genes related to astragaloside IV biosynthesis in *A. membranaceus* after different time treatments. Ethylene on the expression of key genes in the roots of *A. membranaceus*. * and ** represent signifcant correlation in *P* < 0.05 and *P* < 0.01 respectively. “*” and “**” represent the significant differences between control and the corresponding ethephon treatment at the level of 0.05 and 0.01, respectively

### Expression levels of key genes involved in the biosynthesis of astragaloside IV as regulated by ethylene

In order to further investigate the impact of Eth on the expression levels of genes related to astragaloside IV biosynthesis in *A. membranaceus*, ten genes including *AACT*, *HMGS*, *HMGR*, *IDI*, *FPS*, *SS*, *SE*, *CAS*, *CYP88D6* and *CYP93E3* involved in the biosynthetic pathway were selected for analysis after 0 h, 12 h, 3 d and 7 d treatments with 200 µmol·L^− 1^ Eth. Under exogenous Eth treatment, group. The gene expression level of *HMGS* and *CAS*, significantly decreased compared to the control at 12 h and 3th days. The gene expression level of *CYP88D6*, significantly decreased compared to the control at 12 h, 3th days, and 7th days. *FPS*, *SS*, *IDI* and *CYP93E3* demonstrated decreases at 3th days. The *AACT* exhibited initially decreasing, then increasing expression at 7th days. The gene expression level of *HMGR* significantly increased at 12 h, and then its expression decreased compared to the control at 3th days(*P* ≤ 0.05). However, the expression of *SE* significantly decreased at 12 h, and then its expression increased compared to the control at 3th days(*P* ≤ 0.05).

### Correlations of astragaloside IV contents in *A. membranaceus* with the relevant ten key genes expression levels

**Fig. 3:**
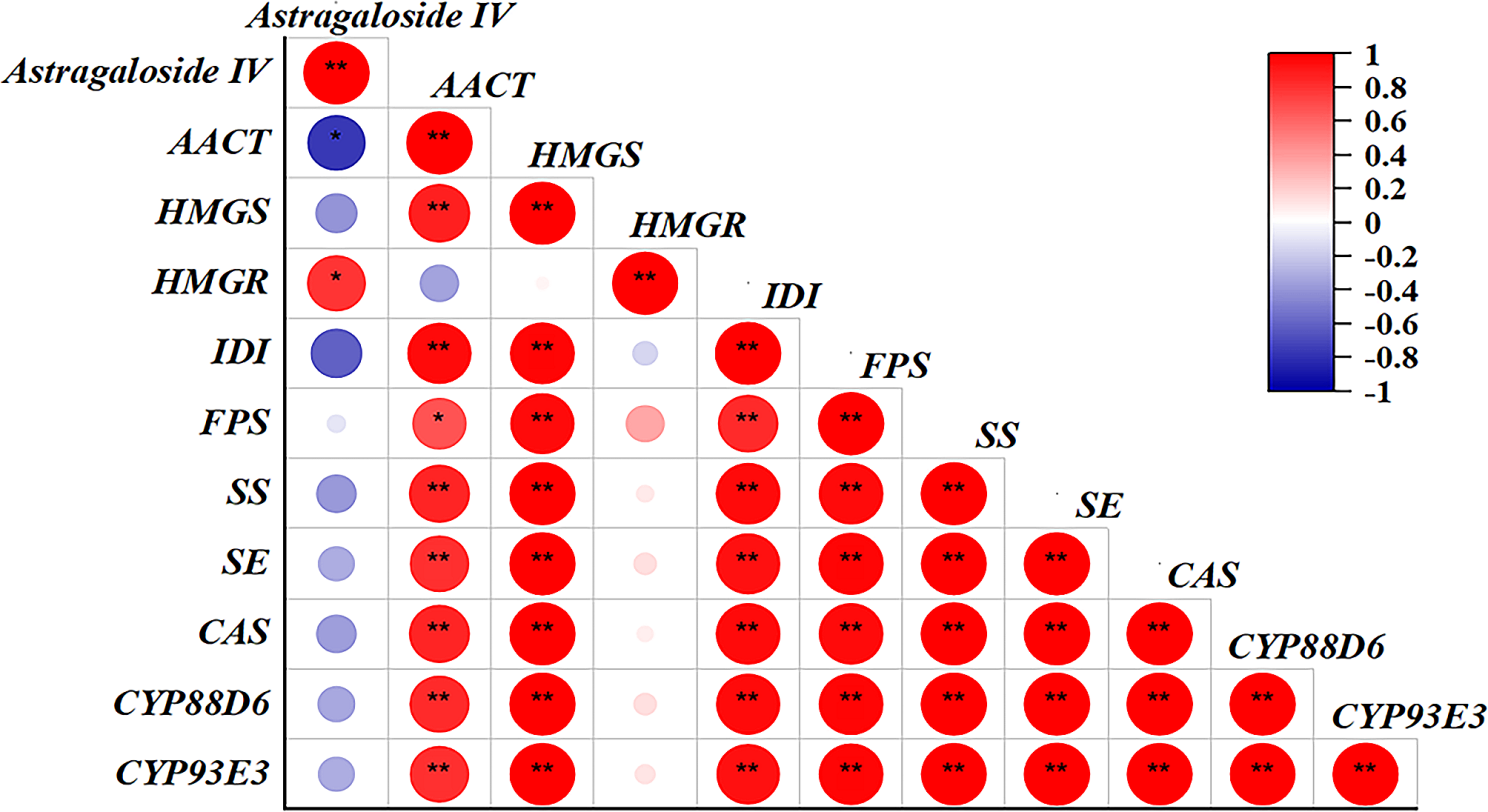
Correlation analysis between astragaloside IV and the ten key genes under ethephon treatment. “*” and “**” represent the significant differences between control and the corresponding ethephon treatment at the level of 0.05 and 0.01, respectively

Correlation analysis was conducted to assess the relationship between astragaloside IV content in *A. membranaceus* roots treated with Eth with the expression levels of key enzyme genes. As depicted in Fig. 3, *FPS* was negatively correlated with astragaloside IV contents at the level of 0.05. Moreover, *HMGR*, *IDI*, *SS* and *CYP93E3* were negatively correlated with astragaloside IV contents at the level of 0.01. Conversely, only *SE* exhibited a significant positive correlation with astragaloside IV content at the level of 0.01. Regarding the five genes that negatively correlated with astragaloside IV contents, four of them *HMGR*, *IDI*, *SS* and *CYP93E3* were also negatively correlated with SE, suggesting SE is a positive role and *HMGR*, *IDI*, *SS* and *CYP93E3* are negative roles in astragaloside IV biosynthesis in *A. membranaceus* as regulated by ethylene.

## Discussion

Astragaloside IV has anti-inflammatory, antioxidant, anti-apoptotic, and tumor-inhibiting biological activities (Liang et al. 2023). Moreover, the content of astragaloside IV serves as an important indicator for assessing the quality of *A. membranaceus* (Zhang et al. 2020). *A. membranaceus* grown hydroponically exhibits significantly high levels of astragaloside IV compared with field cultivation (Chen et al. 2021). Exogenous inducers could modulate the levels of secondary metabolites in plants. Ethylene, as a signaling molecule, plays a regulatory role in plant secondary metabolism (Dubois et al. 2018). For instance, Bae et al. (2006) reported that exogenous 50 µM ethylene treatment significantly enhanced biomass and ginsenoside accumulation in adventitious root of *Panax ginseng* C.A. Meyer (Bae et al. 2006). High concentrations of ethylene stimulate the biosynthesis of saponins in *Calendula officinalis* hairy roots (Markowski et al. 2022). Exogenous ethylene treatment promotes the production of ganoderic acid in *Ganoderma lucidum* (Zhang et al. 2017). In the present study, exogenous application of ethylene significantly increased astragaloside IV contents in *A. membranaceus* roots, and the increase was in a time-dependent manner reaching the peak at 3 h ethylene supply (Fig. 1).

Ethylene plays a crucial role in regulating the changes in active ingredient content in medicinal plants by modulating the expression of key genes involved in secondary metabolism pathways (Tahmasebi et al. 2019). Elucidating the alterations of key genes is essential for controlling the synthesis and accumulation of secondary metabolites (Pan et al. 2015). For instance, ethylene regulates *FPS*, *SS*, and *SE* involved in ginsenoside synthesis, resulting in an increase of ginsenoside contents in *Panax ginseng* C.A. Mey (Rahimi et al. 2015). Similarly, ethylene treatment upregulates key genes of the jasmonate alkaloid synthesis pathway, leading to the accumulation of jasmonate alkaloids in *Catharanthus roseus* (Pan et al. 2018). In *Ganoderma lucidum*, the upregulation of genes such as *HMGR*, *SS*, and *OSC* involved in the biosynthesis of ganoderic acid by ethylene treatment, contributing to increased ganoderic acid accumulation (Xu et al. 2012). Additionally, treatment with an ethylene releaser increases the expression of key genes involved in the biosynthetic pathways of rhynchophylline (RIN) and isorhynchophylline (IRN) in *Uncaria rhynchophylla*, resulting in high RIN and IRN contents in *U. rhynchophylla* leaves (Li et al. 2022). In this study, expression levels of *AACT*, *HMGS*, *HMGR*, *IDI*, *FPS*, *SS*, *SE*, *CAS*, *CYP88D6* and *CYP93E3* were in a time-dependent manner as regulated by ethylene (Fig. 2). Further correlation analysis demonstrated that *FPS*, *HMGR*, *IDI*, *SS* and *CYP93E3* were negatively correlated with astragaloside IV contents, while *SE* was positively correlated with astragaloside IV contents in *A. membranaceus* (Fig. 3). Cultivation of Astragalus membranaceus at high altitude can promote the expression of *SE*, thereby increasing the content of astragaloside IV in Astragalus membranaceus (Wu et al. 2021). Under the regulation of water, the *SE* gene showed a highly significant negative correlation with the content of astragaloside IV and was the main regulatory gene (Wei et al. 2019). Overexpression of SE or downregulation of *FPS*, *HMGR*, *IDI*, *SS* and *CYP93E3* might be the strategies for promoting the astragaloside IV contents in *A. membranaceus*.

**Fig. 4:**
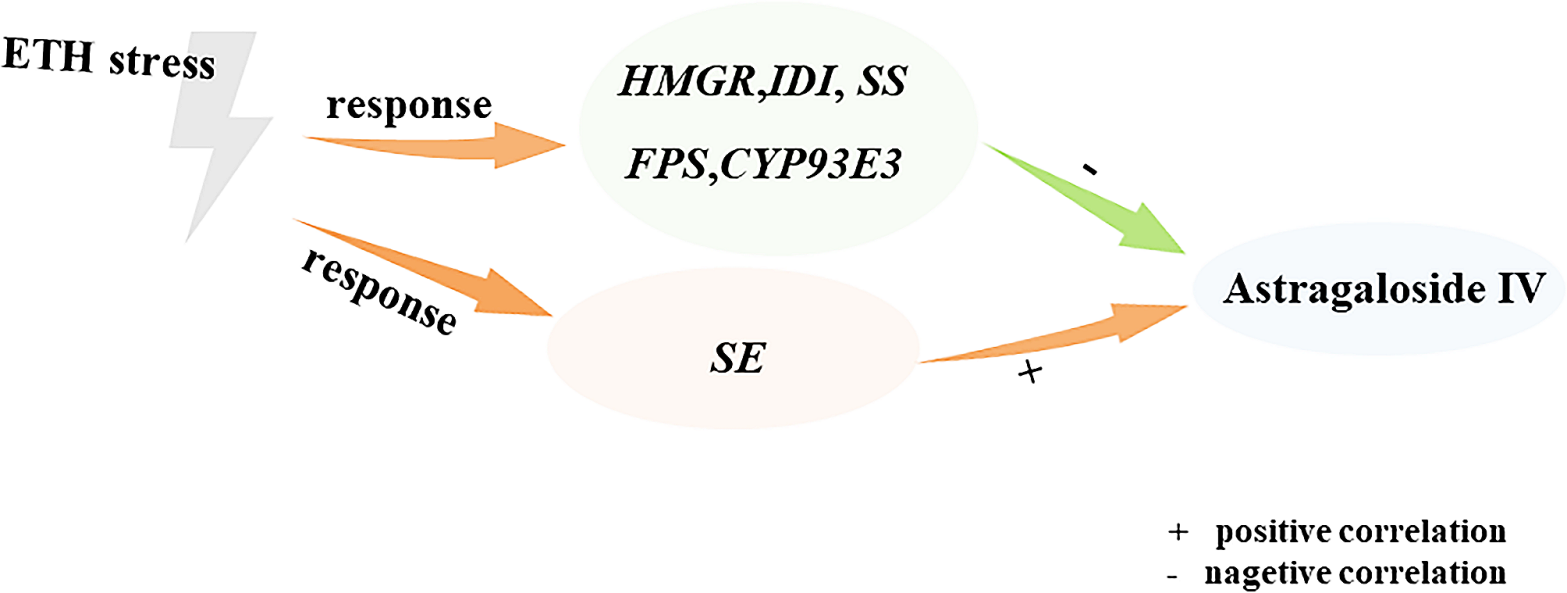
A model of astragaloside IV biosynthesis by genes as regulated by exogenous ethylene

In conclusion, exogenous application ethephon significantly increases astragaloside IV contents in *A. membranaceus* roots grown hydroponically. The astragaloside IV contents as affected by exogenous application ethephon were in a time-dependent manner. In consistent, expression levels of the ten key genes related to astragaloside IV biosynthesis in *A. membranaceus* are also differently affected by exogenous ethephon as the time changes. Correlation analysis exhibits that expression levels of *FPS*, *HMGR*, *IDI*, *SS*, *CYP93E3* are negatively correlated with astragaloside IV contents in *A. membranaceus* roots, whereas *SE* is positively correlated with astragaloside IV contents (Fig. 4). The findings provide new insights into molecular breeding of *A. membranaceus*.

### Electronic supplementary material

Below is the link to the electronic supplementary material.

Supplementary Material 1: Table S Specific primers for RT-qPCR

## Data Availability

The data and material used during the current study are available from the author on reasonable request.
