# Supplementary material for: Exogenous ethephon treatment on the biosynthesis and accumulation of astragaloside IV in Astragalus membranaceus Bge. Var. Mongholicus (Bge.) Hsiao
Source: Bot Stud. 2024 Jul 5;65:16. doi: 10.1186/s40529-024-00426-y (PMC11226570; doi:10.1186/s40529-024-00426-y)
Supplement: Supplementary file 1 — Supplementary Material 1: Table S Specific primers for RT-qPCR [file 40529_2024_426_MOESM1_ESM.docx]

Table S Primers used in this study.

| Primer Name | Sequence 5' → 3' |
| --- | --- |
| 18SRNA-F | TGCAGAATCCCGTGAACCATC |
| 18SRNA-R | AGGCATCGGGCAACGATATG |
| AACT-F | GGTGAGCGGAGAGAAGGCAT |
| AACT-R | CGAGTGCTGGAGCGGTTGTA |
| HMGS-F | TAGAAAAGGCATCCCAAC |
| HMGS-R | CCATAAGAGAATAGTATCACCC |
| HMGR-F | GCCGGCCACCATAAACGA |
| HMGR-R | CGACGGAGAAGAAGAGGGTGAA |
| IDI-F | TGCTGGTGAGGGAGGTTTGAA |
| IDI-R | TCATGTCAGCGACCTCACCAA |
| FPS-F | GGATAACTCTCATACACGCCG |
| FPS-R | GCAGTCTGGAACTCAACCTCAT |
| SS-F | AAGCAGATCCCTCCGGAACC |
| SS-R | ACAGCGTTGCGAAGTTCG |
| SE-F | TGGAACAAGGAACCGTGACATCT |
| SE-R | ACAAAGAGAACGCCTCAAGTTGGA |
| CAS-F | TGGAGATTTCCCACAGCAGGA |
| CAS-R | CAAGTTGCGGCATTTGGTGT |
| DL-AmCYP88D6-F | ACCTCCTGGTGATATGGGATTGCC |
| DL-AmCYP88D6-R | GCACTCGTCTACACATCTCAGGCT |
| DL-AmCYP93E3-F | AACTACCACCAGGACCACCAATT |
| DL-AmCYP93E3-R | AGCTGATGATGCCACTATGACATG |
